# Advanced respiratory monitoring in COVID-19 patients: use less PEEP!

**DOI:** 10.1186/s13054-020-02953-z

**Published:** 2020-05-15

**Authors:** Lisanne Roesthuis, Maarten van den Berg, Hans van der Hoeven

**Affiliations:** Department of Intensive Care Medicine, Radboud University Medical Center, Geert Grooteplein-Zuid 10, 6525 GA Nijmegen, The Netherlands

**Keywords:** Coronavirus disease 2019, Acute respiratory distress syndrome, Positive end-expiratory pressure, Lung compliance, Dead space ventilation, Hyperinflation

To the Editor,

In the majority of coronavirus disease 2019 (COVID-19) patients, respiratory mechanics is different from the “normal” acute respiratory distress syndrome (ARDS) patient. Plateau pressures and driving pressures are often low and respiratory system compliance relatively normal compared to the ARDS patient [1]. Many physicians use high positive end-expiratory pressure (PEEP) for patients with COVID-19 although the potential for recruitment is often low [1, 2]. We fear that the high compliance of the respiratory system in combination with high PEEP will lead to hyperinflation, high dead space, and potentially right ventricular failure.

We have used the following strategy for COVID-19 patients (*N* = 70): after intubation, immediately prone positioning for at least 3 days, using the lowest possible PEEP to obtain adequate oxygenation with FiO_2_ of 50%. We assessed the effects of different PEEP levels on respiratory mechanics and ventilation-perfusion mismatching.

## Methods

Respiratory mechanics was assessed in COVID-19 patients admitted to the Radboud University Nijmegen Medical Center as part of standard patient care. Brief occlusions were performed to assess end-inspiratory and end-expiratory airway and transpulmonary pressures (absolute and elastance ratio method) and to calculate respiratory and lung compliances as previously described [3, 4]. Dead space ventilation was assessed using two methods:

1. The Bohr equation using partial pressure of carbon dioxide in alveolar air (PACO_2_) and mixed expired air (PeCO_2_): (PACO_2_ − PeCO_2_)/PACO_2_. See our previous work for detailed description [5].
2. The Enghoff modification of Bohr’s equation using partial pressure of carbon dioxide in arterial blood (PaCO_2_): (PaCO_2_ − PeCO_2_)/PaCO_2_. Therefore, shunt and diffusion limitations are taken into the equation.

## Results

Advanced respiratory mechanics was assessed in 14 patients (8 males and 6 females, age (mean ± SEM) 67 ± 2 years, body mass index 28.0 ± 0.9 kg/m^2^) between the 19th of March and 2nd of April (Table 1). Compliance of the respiratory system was low (42 ± 3 mL/cmH_2_O) due to a lower than normal lung compliance (61 ± 5 mL/cmH_2_O). However, compared to ARDS patients, lung compliance was relatively high, resulting in low end-inspiratory transpulmonary pressures (12 ± 1 cmH_2_O). Chest wall compliance was slightly lower than normal due to prone positioning in most patients. COVID-19 patients had high dead space ventilation and gas exchange impairment (Bohr 52 ± 3%; Enghoff modification 67 ± 2%).

**Table 1:** Respiratory mechanics

Patient no.	MV days	FiO_**2**_	PaO_**2**_/FiO_**2**_ (mmHg)	PaCO_**2**_ (mmHg)	***P***_**plateau**_ (cmH_**2**_O)	***P***_**drive**_ (cmH_**2**_O)	***P***_**L,e-i**_	***P***_**L,drive**_ (cmH_**2**_O)	***C***_**rs**_ (mL/cmH_**2**_O)	***C***_**L**_ (mL/cmH_**2**_O)	Enghoff (%)	Bohr (%)	Position
1	7	0.50	156	87	22	8	9	5	55	82	–	–	P
2	2	0.45	208	56	24	7	17	18	54	79	–	–	S
	3	0.55	124	57	26	8	–	–	48	–	66	47	S
3	0	0.50	228	44	23	9	17	16	47	62	66	56	S
4	1	0.60	123	44	–	–	–	–	–	–	71	58	P
5	0	0.40	214	48	23	13	9	9	40	54	55	42	P
	1	0.40	278	44	18	10	7	8	50	64	48	38	P
6	1	0.45	143	49	–	–	–	–	–	–	63	40	P
7	1	0.55	183	55	23	14	11	10	36	50	60	42	P
8	1	0.40	176	52	16	8	7	5	56	95	64	51	P
9	0	0.95	98	61	29	12	14	9	38	50	–	–	P
	5	0.60	143	89	27	12	14	9	35	45	72	60	P
10	1	0.80	125	53	21	10	11	7	36	49	66	52	P
11	2	0.55	147	49	21	12	11	10	40	51	69	47	P
12	2	0.75	113	59	25	11	11	8	26	37	69	57	P
	3	0.65	111	47	26	12	11	8	27	40	71	60	P
13	1	0.50	192	67	24	12	10	7	47	76	82	74	P
14	6	0.70	150	62	28	15	15	11	31	43	65	52	P

*C*_*rs*_ compliance of respiratory system, *C*_*L*_ lung compliance, *MV days* days of mechanical ventilation at the time of measurement, *P*_*L,e-i*_ end-inspiratory transpulmonary pressure, *P*_*L,drive*_ transpulmonary driving pressure, *P* prone position, *S* supine position

**Fig. 1:**
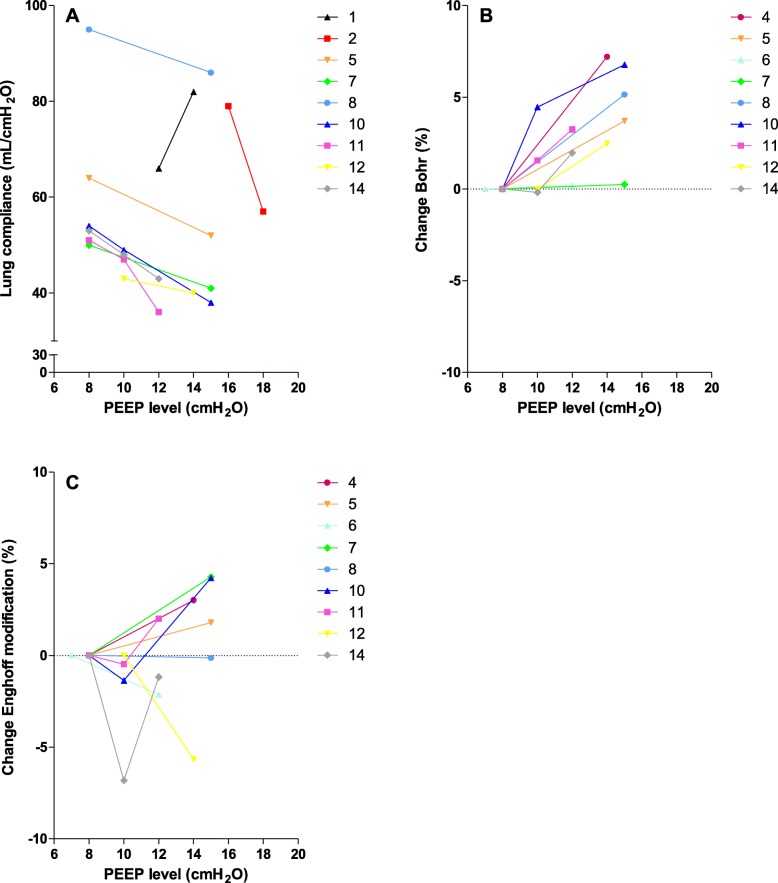
The effects of increasing positive end-expiratory pressure (PEEP) on lung compliance (*N* = 9) and dead space ventilation (*N* = 9). Every patient is depicted with a different symbol and color. **a** Lung compliance decreased with increasing PEEP levels in 8 patients. **b** Dead space ventilation according to Bohr increased in all patients with increasing PEEP levels. **c** In response to higher PEEP levels, dead space ventilation according to Enghoff modification (global gas exchange impairment) increased in 3 patients, first decreased and then increased in 3 patients, decreased in 2 patients, and had no effect in 1 patient

Reducing PEEP resulted in an increase in lung compliance and decrease in dead space ventilation, except for patient 1 (Fig. 1).

## Discussion

We demonstrate that mechanically ventilated patients with COVID-19 have a relatively high lung compliance, high dead space ventilation, and gas exchange impairment. In almost all patients, lung compliance decreased and dead space ventilation increased with increasing PEEP levels.

The decrease in lung compliance and increase in dead space ventilation in response to higher PEEP levels indicate that COVID-19 lesions were not recruited and that higher PEEP levels cause hyperinflation of the more compliant parts of the lung [1]. These results are in accordance with recent findings in COVID-19 patients [2].

When lung compliance increases in response to higher PEEP levels (patient 1), recruitment is likely and PEEP should be set accordingly [1, 2].

All patients responded extremely well to prone positioning, although the exact mechanism is unclear. Redistribution of blood flow seems to be an important mechanism.

In conclusion, we show that higher PEEP levels decrease lung compliance and in most cases increase dead space ventilation, indicating that high PEEP levels probably cause hyperinflation in patients with COVID-19. We suggest using prone position for an extended period of time (e.g., 3–5 days) and apply lower PEEP levels as much as possible.

## Data Availability

The datasets used and analyzed during the current study are available from the corresponding author on reasonable request.

## Abbreviations

ARDS: Acute respiratory distress syndrome
COVID-19: Coronary virus disease 2019
PACO_2_: Partial pressure of carbon dioxide in alveolar air
PaCO_2_: Partial pressure of carbon dioxide in arterial blood
PeCO_2_: Partial pressure of carbon dioxide in mixed expired air
PEEP: Positive end-expiratory pressure
SEM: Standard error of the mean
